# Effects of hempseed (*Cannabis sativa L.*) in diet on growth, gut health, and immunity in broilers

**DOI:** 10.1080/01652176.2024.2367214

**Published:** 2024-06-18

**Authors:** Bushra Sana, Naila Chand, Shabana Naz, Ibrahim A. Alhidary, Rifat Ullah Khan, Shamsuddin Shamsi, Caterina Losacco, Vincenzo Tufarelli

**Affiliations:** a Department of Poultry Science, Faculty of Animal Husbandry and Veterinary Sciences, The University of Agriculture, Peshawar, Khyber Pakhtunkhwa, Pakistan; b Department of Zoology, Government College University, Faisalabad, Pakistan; c Department of Animal Production, College of Food and Agriculture Science, King Saud University, Riadh, Saudi Arabia; d College of Veterinary Sciences, Faculty of Animal Husbandry & Veterinary Sciences, The University of Agriculture, Peshawar, Pakistan; e Department of Precision and Regenerative Medicine and Jonian Area (DiMePRe-J), Section of Veterinary Science and Animal Production, University of Bari ‘Aldo Moro’, Valenzano, Italy

**Keywords:** Broiler, *Cannabis sativa*, gut pH, histology, immunity

## Abstract

The aim of the present study was to assess the effects of different levels of hempseed (HS) on growth performance, immunity and gut health in broiler chickens. A total of 192 Hubbard broiler chicks were divided into four groups and fed HS as follow: control (HS0), HS 10% (HS-10), HS 15% (HS-15) and HS 20% (HS-20). The study on HS supplementation in broilers revealed no significant impacts on feed intake during the starter (*p* = .2294) and finisher phases (*p* = .2294), or overall (*p* = .0944), though numerical increases were noted with higher HS levels. Body weight gain showed no significant influence in the starter and finisher phases, with overall weight gain also not significantly different (*p* = .0944), but numerically higher with increased HS. Feed conversion ratio was unaffected in the starter (*p* = .6986) and finisher phases (*p* = .6425), and overall (*p* = .2218). Dressing percentage (*p* = .1062) and mortality (*p* = .1631) were not significantly altered, but HS-20 had the highest dressing percentage and lowest mortality numerically. White blood cell counts increased significantly (*p* = .0377), especially in HS-15 and HS-20 groups. IgM and IgG production was higher in HS-20 on day 28 (*p* = .021). Gut pH (*p* > .05) and intestinal histomorphology (*p* > .05) were not significantly affected, although villus height increased numerically with higher HS levels. These results suggest potential benefits of HS, especially at higher inclusion levels. In conclusion, the obtained results indicated that HS incorporation into the diet of broilers did not affect the growth performance and gut health; however, the immune responses were significantly higher at 15 and 20% levels.

## Introduction

Recently, groundbreaking creative approaches such as organic poultry farming have captured the interest of nutrition scientists (Farooq et al. 2022; Imtiaz et al. 2023; Subhan et al. 2023; Hafeez et al. 2024). Within the realm of organic production, there exists a significant demand for knowledge concerning feed quality, feeding techniques, and optimal feed utilization (Jakobsen and Hermansen 2001; Foroutankhah et al. 2019; Vispute et al. 2019; Ahmad et al. 2023; Hafeez et al. 2023). *Cannabis sativa* L. is an annual, dioecious plant with lush green foliage (Adams and Martin 1996; Shahid et al. 2015). It thrives in low-temperature conditions, which makes it feasible to cultivate when growing other oil seeds proves to be challenging (Karimi and Hayatghaibi 2006; Shariatmadari 2023). The primary products derived from *Cannabis sativa* include whole seeds, seed meal, hulled seeds, oil, fiber, and hashish (Adams and Martin 1996; Callaway 2004). Whole hemp seeds (HS) have been demonstrated to possess a metabolizable energy (ME) content of 18.0 MJ/kg for pigeons (Hullar et al. 1999). The seeds also exhibited protein levels ranging from 20% to 24% (Hullar et al. 1999; Fortenbery and Bennett 2004; Raza et al. 2016), as well as containing 33% ether extract (EE) and 35% carbohydrates, primarily in the form of fiber (Khan et al. 2010).

Around 65%–70% of the overall expenses in poultry production are attributed to feed costs. Typically, poultry feeding relies on corn-soy based diets. However, there has been a quest for alternative raw materials to meet the substantial energy and protein requirements of birds (Gul and Alsayeqh 2023; Mohamed and Hassan 2023). This pursuit may stem from concerns over anti-nutritional factors in conventional raw materials, as well as their high costs and utilization limitations (Andualem 2023). The HS protein lacks trypsin inhibitors and oligosaccharides commonly found in soybeans, which are known to cause stomach discomfort and flatulence (Shaghaghian et al. 2022). This unique quality has led to the historical utilization of HS in traditional medicine for addressing issues related to gas and indigestion (Eriksson and Wall 2012). The premium protein in HS is easily digestible and contain significant quantities of all essential amino acids, providing crucial nutritional value (Callaway 2004). Notably, HS contains higher levels of arginine in comparison to others parts of the plant, a feature that distinguishes it from the other plant origin protein (Callaway 2004). Industrial HS exhibit a low tetrahydrocannabinol (THC) content (∼0.3%), which is known to trigger appetite (Hampson et al. 2000; Koch 2001; Konca et al. 2014). The THC metabolite is cannabidiol (CBD), which is recognized for its immunomodulatory and antioxidative properties (Korhonen and Pihlanto 2003). In the last few years, the inclusion of phytobiotics and other feed additives in broiler diet has been increased immensely to improve growth and health of broilers (Landy et al. 2020; Khan et al. 2022; Nuriyasa et al. 2022; Orinetha et al. 2022; Mickdam et al. 2023).

Recent studies on the supplementation of hemp seeds (HS) and hempseed cake (HSC) in livestock diets reveal consistent benefits in fatty acid profiles and milk production across various animal species. Mierlita (2016, 2018) demonstrated that HS supplementation in ewes’ diets improved milk fat content and fatty acid composition. This effect was supported by Cremonesi et al. (2018), who observed changes in the rumen biohydrogenation pathway in goats fed HS, leading to increased beneficial fatty acid intermediates. Similarly, Karlsson et al. (2010) found that moderate HSC supplementation in dairy cows enhanced milk yield, though higher levels reduced protein conversion efficiency, indicating an optimal inclusion threshold. Hessle et al. (2008) and Turner et al. (2008) further supported these findings by showing that HSC can serve as an effective alternative protein source without negatively impacting growth or carcass quality in calves and steers. Collectively, these studies suggest that incorporating hemp products into livestock diets can enhance milk quality and yield, improve fatty acid profiles, and serve as a sustainable protein source, though optimal inclusion levels must be carefully managed to maximize benefits.

Studies on HSC and HS supplementation in poultry diets provide valuable insights into its impacts. Stastník et al. (2019) found that adding 15% HSC to broiler diets reduced live weight due to higher fiber content, while 5% HSC had no impact on weight. Carcass weight and meat composition remained unaffected regardless of HSC level. Similarly, Neijat et al. (2014) reported that feeding laying hens with up to 30% HS did not affect overall performance metrics. These findings indicate that while moderate HS or HSC inclusion does not adversely affect poultry performance, higher HSC levels may reduce broiler weight due to increased dietary fiber. Optimal inclusion levels must be managed to avoid negative impacts on growth while leveraging the nutritional benefits of hemp products. The legalization of hemp cultivation has spurred consumer interest in its diverse products. Despite initial challenges, such as regulatory compliance and market fluctuations, hemp farming offers promising economic opportunities. As consumer demand grows, the industry is poised for further expansion. Hempseed cake serves as a cost-effective alternative protein source for poultry and livestock, offering nutritional benefits while being economically viable for farmers.

As hemp production continues to rise, its by-products are becoming increasingly prevalent. The recent legalization of hemp cultivation has sparked a surge in research focused on exploring the integration of hemp-derived products in animal dietary applications. However, investigations into the incorporation of hemp during the growing stage of poultry have been limited. We hypothesize that the inclusion of HS could potentially influence nutrient intake, consequently impacting the performance and overall health of broilers. Recognizing the implications of dietary composition on the functionality of the digestive tract holds paramount importance. The aim of the present study was to investigate different inclusion levels of HS in the diet of broiler to evaluate the effects on growth performance, immunity, gut pH and intestinal histology.

## Materials and methods

### Experimental animals and diets

Newly hatched Hubbard broiler chicks (*n* = 192) possessing consistent body weights were selected in a random manner. Each chick was individually weighed and then sorted into four distinct treatment groups. Each treatment group comprised of six separate replicates, and within these replicates, there were eight birds per replicate. Over a span of 35 days, these chicks were raised within battery brooder cages, all while adhering to consistent and uniform management protocols. During this 35-day rearing period, a uniform approach was taken in the management of these chicks within the battery brooder cages. Additionally, an assortment of six experimental diets was developed for the chicks. These diets were formulated to encompass varying quantities of HS, resulting in a total of eight diets when including the basal diet. Each experimental diet was designed to include different proportions of sun-dried HS, namely HS-0, 10, 15, and 20 representing 0, 10, 15 and 20% inclusion of HS in the offered feed respectively in mash form. Table 1 (starter phase) and Table 2 (Finisher phase) provide a comprehensive overview of the constituents and nutritional profile of the basal diet utilized throughout the starter phase and the finisher phase according to NRC (1994). Mortality was recorded as it occurred.

**Table 1.** Basal diet composition of experimental diets during the starter phase (1–21 days).

Ingredients (%)	Control	HS-10	HS-15	HS-20
Corn	54.0	50.90	48.80	45.50
Soybean meal (47% CP)	41.80	34.90	32.0	30.95
Hemp seeds	0.00	10.00	15.00	20.00
Limestone	1.20	1.20	1.20	1.20
Dicalcium phosphate	2.10	2.10	2.10	2.10
Salt	0.30	0.30	0.30	0.30
Vitamin-mineral premix^1^	0.50	0.50	0.50	0.50
L-Lysine	0.02	0.02	0.02	0.02
DL-Methionine	0.13	0.13	0.13	0.13
Nutrients analysis				
Crude protein	22.00	22.00	22.00	22.00
Metabolizable energy (kcal/kg)	3000	3000	3000	3000
Calcium (%)	0.90	0.90	0.90	0.90
Available phosphorus (%)	0.35	0.35	0.35	0.35
Lysine (%)	1.30	1.30	1.30	1.30
Methionine (%)	0.50	0.50	0.50	0.50
Methionine + Cysteine (%)	0.80	0.80	0.80	0.80

HS-0: (Control) no hemp seeds inclusion; HS-10 hemp seeds inclusion at the rate of 10%; HS-15: hemp seeds inclusion at the rate of 15%; HS-20: hemp seeds inclusion at the rate of 20%.

^1^ Vitamin-mineral premix (per kilogram of diet) – Vitamin A 14,000 IU; Vitamin K 6 mg; Vitamin D3 1400 IU; Vitamin B2 7 mg; Vitamin B1 4 mg Vitamin B6; 4 mg; Vitamin B12 0.04 mg; Biotin 0.2 mg; Niacin 34 mg; folic acid 1.0 mg; calcium D- panthotenate 14.0 mg; Manganese 80 mg; coline chloride 400 mg; Zinc 50 mg; Iron 35 mg; Iodine 2 mg; Copper 5.0 mg; Cobalt 0.04 mg.

**Table 2.** Basal diet composition of experimental diets during the finisher phase (22–35 days).

Ingredients (%)	Control	HS-10	HS-15	HS-20
Corn	54.0	50.1	47.00	45.20
Soybean meal (47% CP)	41.40	35.35	33.5	30.35
Hemp seeds	0.00	10.0	15.0	20.0
Limestone	1.20	1.20	1.20	1.20
Dicalcium phosphate	2.10	2.10	2.10	2.10
Salt	0.30	0.30	0.30	0.30
Vitamin-mineral premix^1^	0.50	0.50	0.50	0.50
L-Lysine	0.17	0.2	0.2	0.15
DL-Methionine	0.25	0.25	0.24	0.20
Nutrients analysis				
Crude protein (%)	20.00	20.00	20.00	20.00
Metabolizable energy (kcal/kg)	3000	3000	3000	3000
Calcium (%)	0.90	0.90	0.90	0.90
Available phosphorus (%)	0.35	0.35	0.35	0.35
Lysine (%)	1.15	1.15	1.15	1.15
Methionine (%)	0.40	0.40	0.40	0.40
Methionine + Cysteine (%)	0.75	0.75	0.75	0.75

HS-0: (Control) no hemp seeds inclusion; HS-10: hemp seeds inclusion at the rate of 10%; HS-15: hemp seeds inclusion at the rate of 15%; HS-20: hemp seeds inclusion at the rate of 20%.

^1^ Vitamin-mineral premix (per kilogram of diet) – Vitamin A 14,000 IU; Vitamin K 6 mg; Vitamin D3 1400 IU; Vitamin B2 7 mg; Vitamin B1 4 mg Vitamin B6; 4 mg; Vitamin B12 0.04 mg; Biotin 0.2 mg; Niacin 34 mg; folic acid 1.0 mg; calcium D- panthotenate 14.0 mg; Manganese 80 mg; coline chloride 400 mg; Zinc 50 mg; Iron 35 mg; Iodine 2 mg; Copper 5.0 mg; Cobalt 0.04 mg.

### Growth performance

Each dietary regimen was provided in measured amounts and was available ad libitum twice daily (Javed et al. 2021). The remaining feed was weighed daily to determine the feed intake. The individual weights of the birds were initially recorded on the first day, followed by weekly measurements throughout the experimental phase. These periodic measurements allowed for the assessment of both weekly and cumulative body weight gains. At the conclusion of each experimental period, the feed conversion efficiency was determined by dividing the amount of feed consumed by the increase in body weight. On day 35, eight birds from each dietary treatment group, selected randomly based on the average weight of the group, were slaughtered. The process involved the removal of the skin, head, feet, and internal organs. Subsequently, the dressing percentage, which indicates the proportion of usable meat in relation to the initial live weight, was measured.

### White blood cells (WBCs) count

At the conclusion of the experiment, three birds from each replicate were selected for slaughter. Subsequent to slaughter, their blood was collected through decapitation, with the blood samples were being collected in tubes containing EDTA to serve as an anticoagulant. This collected blood was utilized for the blood smears, employing two glass slides, one of which possessed cut edges. The smears were meticulously prepared and subjected to a drying process before undergoing staining using the May-Grunwald-Giemsa stain. The resultant stained smears were then subjected to a microscopic examination using a Nikon YS 100 microscope to count WBCs using Neubauer chamber (Hassan et al. 2022, 2023). Total leucocyte count was calculated using the following formula

Total WBCs = counted cells ×10 × dilution factor/total area counted = total WBC count/µL

### Evaluation of IgM and IgG profile

Sheep red blood cells (SRBC), used as a non-pathogenic antigen, were employed to assess the humoral immune response in broiler chickens on day 28 and 35. A total of eight birds from each treatment group (two birds per replicate) were identified with dye markers. On the 21st day of age, these birds were administered an injection of 0.1 ml of a 5% SRBC suspension into the brachial vein. Following the initial challenge, a secondary immune response was prompted by administering a booster injection of the 5% SRBC suspension to the birds seven days later. Blood samples were collected on the seventh day subsequent to each inoculation. These blood samples were left at room temperature for a period of two hours, promoting the formation of clots to yield sera. Serum samples were subjected to testing for immunoglobulin G (IgG) and immunoglobulin M (IgM) using the 2-mercaptoethanol sensitive (MERC-sensitive) technique, as outlined by Zarghi et al. (2022). In brief, serum was carefully pipetted into microcentrifuge tubes and subjected to heat inactivation by immersing in a 56 °C water bath for a period of 30 min.

Following inactivation, 50 mL of phosphate-buffered saline (PBS) was dispensed into the first row of wells within a 96-well micro-titration plate. Subsequently, 50 mL of the inactivated serum was added to the same wells. The plates were securely sealed and then incubated at a temperature of 37 °C for a duration of 30 min. After this incubation period, an addition of 50 mL of PBS was made to the remaining 11 wells in every row. A progressive serial dilution of the samples was performed across successive rows. Furthermore, 50 mL of a 2.5% SRBC suspension was added to each well. The plates were once again sealed and subjected to an additional incubation period of 30 min. To assess the antibody titers for both IgM and IgG, 50 mL of 2-mercaptoethanol (2-ME) was introduced into the wells of the first row. The resulting titers were determined by holding the plates over a mirror with a source of light, facilitating the observation of agglutination wells. The antibody titers were then presented as Log_2_ of the reciprocal of the final dilution showing observable agglutination.

### pH of the digesta contents

At day 35 of age, six chickens of nearly the same body weight were selected from each dietary treatment, slaughtered and eviscerated. The components of digestive tract crop, gizzard, proventiculus, gizzard, duodenum and jejunum were emptied through delicate compression. The pH of the contents of the individual segments were measured with a pH meter (Cherian et al. 2013). In brief, digesta samples were collected from various sections of the gastrointestinal tract, and their pH was measured using a calibrated digital pH meter. The pH values were recorded and analyzed to assess the impact of dietary treatments.

### Intestinal histology

At the 35-day of the experiment, six healthy birds from each treatment group were selected. Using Meckel’s diverticulum as a reference point at the junction between the jejunum and ileum, a section of the jejunum measuring between 0.5 and 1 cm was carefully collected from each bird. This collected segment was then immersed in a solution of 10% formalin to preserve the tissue, with the precaution of removing intestinal contents without causing damage to the mucosal lining. The preserved samples were kept until they were ready to be processed for the creation of histological slides. Subsequently, the prepared slides were examined using a microscope, using low magnification (10×). The objective of this examination was to assess the characteristics of the jejunal villi. By utilizing Motic Real Imaging Software, the height and mean width of these villi were computed. This process allowed for a detailed evaluation of the structural aspects of the jejunum’s villi and their potential variations among the different dietary treatment groups. Crypt depth was measured from the base of the intestinal crypt to the surface level of the surrounding mucosa, while villus height refers to the distance from the tip of the villus to its base at the crypt-villus junction.

### Statistical analysis

The data underwent processing using Microsoft Excel (USA) and Statistica version 12.0 (CZ). For the statistical analysis, a one-way analysis of variance (ANOVA) was employed. To ascertain significant differences with confidence, Scheffe’s test was applied. A significance level of *p* < .05 was utilized to determine statistically meaningful distinctions between the groups.

## Results

Effect of HS supplementation on the feed intake in broilers is shown in Table 3. Feed intake in starter and finisher phases was not influenced (*p* = .22) by the dietary treatments; however, numerically feed intake in the finisher phase was linearly increased with increasing the inclusion level of HS in the diets of treatment groups. Similarly, feed intake throughout the experimental period (overall feed intake) was the same (*p* = .09) in all groups.

**Table 3.** Mean feed intake (g) in broilers fed with different levels of hemp seed in the ration.

Groups	Starter phase	Finisher phase	Overall
HS-0	1236.7 ±7.6	2227.7 ± 2.5	3472.3 ± 2.5
HS-10	1237.7 ± 2.5	2231.3 ± 2.5	3472.0 ± 2.6
HS-15	1240.7 ± 2.5	2234.3 ± 5.0	3473.0 ± 2.0
HS-20	1244.3 ± 3.1	2239.7 ± 7.6	3475.0 ± 6.6
*P*-value	.2294	.0761	.0944

HS-0: (Control) no hemp seeds inclusion; HS-10: hemp seeds inclusion at the rate of 10%; HS-15: hemp seeds inclusion at the rate of 15%; HS-20: hemp seeds inclusion at the rate of 20%.

Effect of HS supplementation on body weight gain in broilers is shown in Table 4. During starter and finisher phase, weight gain was not significantly influenced by supplementation of HS; however, weight gain was linearly increased with increasing the inclusion level of HS in the diet. Similarly, overall weight gain did not vary significantly by HS supplementation in the ration; however, numerically overall weight gain was linearly increased with increasing the inclusion levels of HS in the diet of treatment groups.

**Table 4.** Mean weight gain (g) in broilers fed with different levels of hemp seed in the ration.

Groups	Starter phase	Finisher phase	Overall
HS-0	698.00 ± 2.6	1145.0 ± 5.0	1849.0 ± 6.0
HS-10	699.67 ± 2.5	1151.0 ± 2.1	1852.7 ± 4.0
HS-15	701.33 ± 1.5	1154.3 ± 2.0	1856.7 ± 8.7
HS-20	705.67 ± 1.2	1163.0 ± 1.0	1864.7 ± 7.0
*P*-value	.1057	.0995	.0654

HS-0: (Control) no hemp seeds inclusion; HS-10: hemp seeds inclusion at the rate of 10%; HS-15: hemp seeds inclusion at the rate of 15%; HS-20: hemp seeds inclusion at the rate of 20%.

Effect of HS supplementation on FCR in broilers is shown in Table 5. During starter phase, no difference (*p* = .69) was observed in feed conversion ratio among the treatment groups. Feed conversion ratio in finisher phase was also not different (*p* = .64) among the treatment groups. Similarly, chicks in all the treatment groups did not differ significantly (*p* = .22) in feed conversion ratio throughout the experimental period.

**Table 5.** Mean feed conversion ratio in broilers fed with different levels of hemp seed in the ration.

Groups	Starter phase	Finisher phase	Overall
HS-0	1.77 ± 0.1	1.95 ± 02	1.88 ± 0.3
HS-10	1.76 ± 0.3	1.94 ± 01	1.87 ± 0.2
HS-15	1.77 ± 0.1	1.94 ± 0.3	1.87 ± 0.1
HS-20	1.76 ± 0.2	1.93 ± 0.1	1.86 ± 0.3
*P*-value	.6986	.6425	.2218

HS-0: (Control) no hemp seeds inclusion; HS-10: hemp seeds inclusion at the rate of 10%; HS-15: hemp seeds inclusion at the rate of 15%; HS-20: hemp seeds inclusion at the rate of 20%.

Effect of HS supplementation on the dressing percentage and mortality in broilers is shown in Table 6. Throughout the experiment, dressing percentage was not influenced (*p* = .10) by the dietary treatments. Even though, group HS-20 had numerically high value for dressing percentage, but there was no statistical difference among the treatment groups. Similarly, mortality was not deferred significantly (*p* = 0.16) among the treatment groups. However, group HS-20 which have highest inclusion of HS had the least numerical value for mortality. Contrarily, group HS-0 which was control group (having no inclusion of HS) showed highest numerical value for mortality. This study showed that white blood cells count was influenced (*p* = 0.03) by supplementation of HS in the diet of broiler chicks. The minimum white blood cells count was recorded in group HS-0 and HS-10, while white blood cells count in groups HS-15 and HS-20 was greater (*p* < 0.03) among the treatment groups.

**Table 6.** Mean dressing percentage, white blood cells count and mortality in broilers fed with different levels of hemp seed in the ration.

Groups	Dressing percentage	WBCs (×10^3^/µl)	Mortality %
HS-0	69.7 ± 0.4	1.54 ± 0.52^c^	1.33 ± 0.6
HS-10	70.5 ± 1.4	1.36 ± 0.42^c^	1.00 ± 0.5
HS-15	70.8 ± 0.7	1.76 ± 0.50^b^	0.67 ± 0.2
HS-20	71.7 ± 0.6	1.97 ± 0.29^a^	0.33 ± 0.3
*P*-value	.1062	.0377	.1631

Mean values within a column bearing different superscripts differ significantly (*p* < .05). WBCs: white blood cells; HS-0: (Control) no hemp seeds inclusion; HS-1: hemp seeds inclusion at the rate of 10%; HS-15: hemp seeds inclusion at the rate of 15%; HS-20: hemp seeds inclusion at the rate of 20%.

The effect of HS supplementation on the antibody response to sheep RBCs in broilers is shown in Table 7. IgM and IgG production was significantly (*p* = 0.021) higher in HS-20 on day 28 of the experiment compared to the control. Level of Ig-G and Ig-M did not differ significantly between the control and treatment groups on day 35.

**Table 7.** Mean antibodies response to sheep red blood cells in broilers fed with different levels of hemp seed in the ration.

Groups	IgM-titre		IgG-titre	
	Day-28	Day-35	Day-28	Day-35
HS-0	0.66 ± 0.57^bc^	1.0 0± 0.00	0.67 ± 0.57^c^	0.66 ± 0.57
HS-10	1.00 ± 0.00^b^	1.0 0± 0.00	2.00 ± 1.00^c^	0.67 ± 0.57
HS-15	2.00 ± 1.00^ab^	1.33 ± 0.00	3.00 ± 1.00^b^	1.00 ± 1.00
HS-20	3.00 ± 1.00^a^	1.33 ± 0.00	6.00 ± 1.00^a^	2.00 ± 1.00
*P*-value	.02	.59	.01	.026

Mean values within a column bearing different superscripts differ significantly (*p* < .05).

HS-0: (Control) no hemp seeds inclusion; HS-10: hemp seeds inclusion at the rate of 10%; HS-15: hemp seeds inclusion at the rate of 15%; HS-20: hemp seeds inclusion at the rate of 20%.

Effect of HS supplementation on the gut pH in broilers is shown in Table 8. In this study, gut pH was not influenced (*p* > .05) by the supplementation of HS in the diet of broiler chicks.

**Table 8.** Mean pH of digesta in different compartments in broilers fed with different levels of hemp seed in the ration.

Group	Crop	Proventiculus	Gizzard	Duodenum	Jejunum
HS-0	5.51 ± 0.02	3.00 ± 0.10	3.10 ± 0.10	5.60 ± 0.10	6.28 ± 0.66
HS-10	5.49 ± 0.01	3.00 ± 0.20	3.00 ± 0.10	5.50 ± 0.10	6.60 ± 0.06
HS-15	5.49 ± 0.10	3.00 ± 0.30	3.07 ± 0.25	5.46 ± 0.15	6.50 ± 0.08
HS-20	5.49 ± 0.21	2.90 ± 0.10	2.93 ± 0.05	5.53 ± 0.057	6.53 ± 0.08
*P*-value	.083	.89	.54	.51	.64

HS-0: (Control) no hemp seeds inclusion; HS-10: hemp seeds inclusion at the rate of 10%; HS-15: hemp seeds inclusion at the rate of 15%; HS-20: hemp seeds inclusion at the rate of 20%.

**Figure 1.**
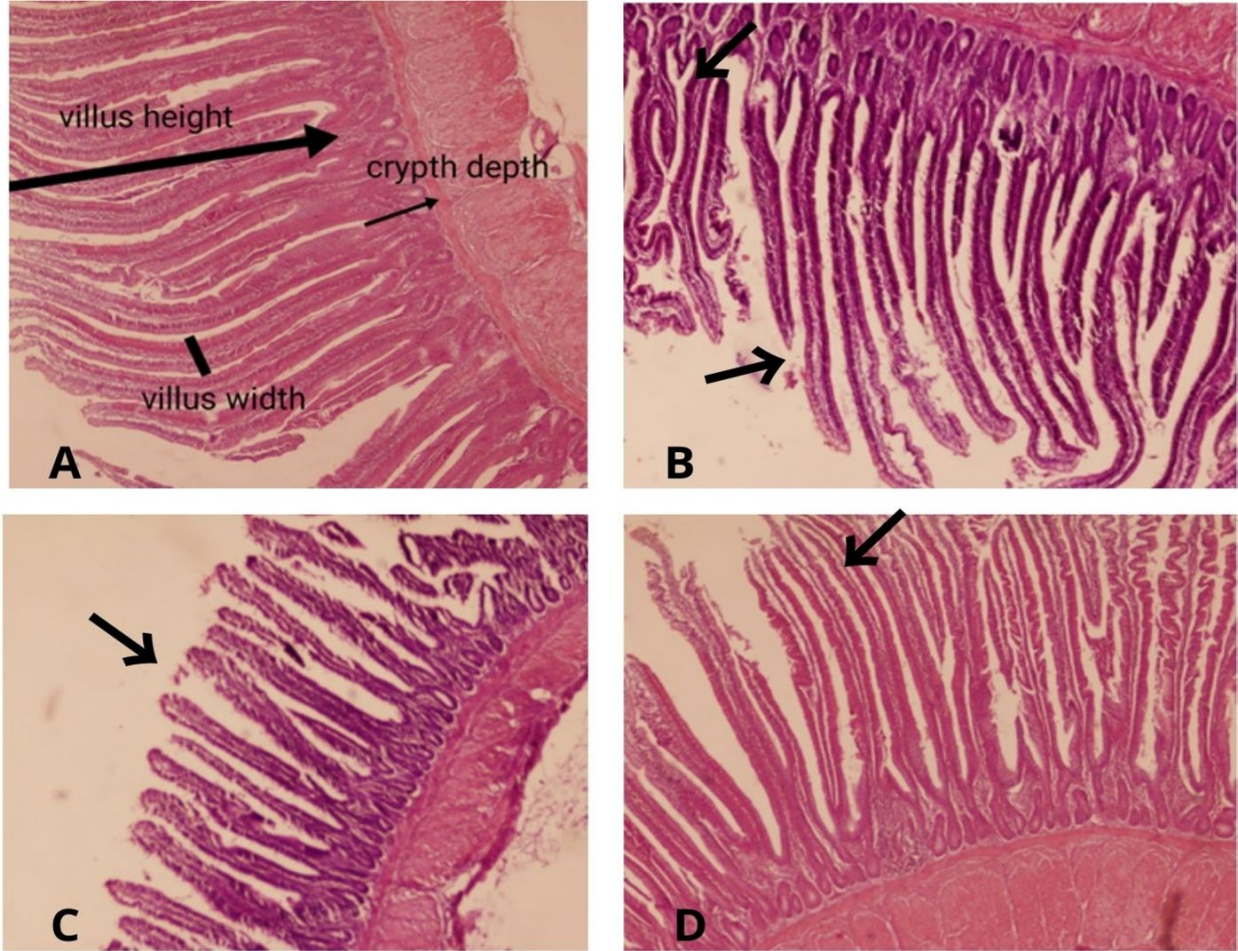
Effects of different levels of cannabis seeds on jenunum histomorphology in broilers HS-0 (a), HS-10 (B), HS-15 (C) and HS-20 (D); arrows indicate intact villi in the control and treatment groups.

Effect of HS supplementation on the jenunum histomorphology (villus height, villus width, crypt depth and their ratio) in broilers is shown in Table 9 and depicted in Figure 1. In this study, jenunum histomorphology was statistically not influenced (*p* > .05) by the supplementation of HS in the diet of broiler chicks. Although the difference among the treatment groups was not significant statistically, numerically, villus height tended to increased linearly with increasing the level of HS in the diets of the treatment groups. Villus height was maximum in group HS-20 (highest level of HS) while the height was minimum in the chicks of group HS-0. Further, the addition of HS did not vary the microstructures of the villi in all groups.

**Table 9.** Mean jenunum histomorphology in broilers fed with different levels of hemp seed in the ration.

Groups	Villus height (um)	Villus width (um)	Crypt depth (um)	Villus height crypt depth ratio
HS-0	1243 ±3.2	168 ± 4.54	193 ± 5.51	6.33 ± 1.21
HS-10	1252 ± 3.9	171 ±5.48	195 ± 4.03	6.54 ± 1.32
HS-15	1270 ± 2.3	170 ±5.45	196 ± 4.21	6.42 ± 1.36
HS-20	1286 ±4.1	180 ±4.92	197 ± 4.35	7.31 ± 1.43
*P*-value	.074	1.34	2.23	2.98

HS-0: (Control) no hemp seeds inclusion; HS-10: hemp seeds inclusion at the rate of 10%; HS-15: hemp seeds inclusion at the rate of 15%; HS-20: hemp seeds inclusion at the rate of 20%.

## Discussion

This study examined the impact of HS supplementation on broiler performance, showing no significant differences in feed intake, body weight gain, feed conversion ratio, dressing percentage, and mortality across dietary treatments. Notably, HS-20 supplementation improved white blood cell count and antibody response, suggesting potential benefits for broiler health and immunity, with implications for enhanced disease resistance and reduced drug use. Our findings align with several other studies, including those conducted by Eriksson and Wall (2012), Afzali et al. (2015), Mahmoudi et al. (2015), Stastník et al. (2015), Vispute et al. (2019) and Skřivan et al. (2020) which reported that the incorporation of HS into broiler feed had no significant impact on performance indicators. In contrast, Barani et al. (2015) noted a significant decrease in feed intake and body weight of broilers when HS was added at a 10% dose rate. Conversely, in a study by Khan et al. (2010), it was noted that the average body weight gain and dressing percentage of chickens exhibited a significant enhancement in a group that received a 20% cannabis supplement, as opposed to the control group, by the conclusion of the experiment. However, Stastník et al. (2015) demonstrated that the inclusion of HS expellers at a 15% dosage had an adverse impact on chicken growth. This was evident in the significantly lower final body weight (at 37 days of age) when HS cakes were incorporated into the feed mixture. Moreover, a higher proportion (15%) of HS cakes also led to a worsened FCR. Feed intake, a crucial factor affecting the performance of poultry, is intricately tied to their appetite condition. Remarkably, the presence of cannabinoid receptors in the brain implies that cannabis consumption can potentially impact appetite, encompassing eating patterns and the control of body weight. However, there exists a notable disparity in reported feed intakes across the aforementioned studies. For instance, Skrivan et al. (2020) documented an increase in feed intake among broilers fed with HS. On the other hand, Mahmoudi et al. (2015) found no discernible effect on feed intake within the HS-fed group. In contrast, Khan et al. (2010) reported that the HS-fed broilers displayed lower feed intake in comparison to the control group. In the study conducted by Bahar et al. (2014), initial observations revealed no alterations in feed intake among HS-fed broilers up to the conclusion of the second week; however, a decrease was noted by the sixth week. It is evident that the diverse levels of HS employed across various experiments present challenges when attempting to effectively compare results. This variation in HS inclusion levels further underscores the complexity of understanding the interaction between HS supplementation and poultry performance. Konca et al. (2014) proposed that the inclusion of hempseed in the diet may lead to an excessive increase in certain amino acid content. This could potentially result in an imbalance of amino acid ratios, which may have an antagonistic effect and reduce the bioavailability of amino acids. Second, while higher levels of hempseed may provide a greater availability of protein and energy, the elevated cellulose content could potentially have a detrimental impact on growth performance. This is why, despite the increased inclusion of hempseed, the growth performance of broilers did not show a positive effect (Konca and Beyzi 2012). The precise cause of the fluctuations in broiler growth performance in response to varying doses of HS is not completely understood and warrants further investigation. However, it can be inferred that the growth performance of broilers receiving HS supplementation is influenced by a range of factors, including the form, dosage, and duration of HS supplementation, as well as the broiler strain and other experimental conditions.

In the current study, no significant change was observed in the pH of the contents of guts and the histology of jejunum in the treatment groups. Similar observations were recorded in the study of Vispute et al. (2019) fed different levels of HS to broiler chickens. However, studies are scarce on the effect of HS incorporation in ration on intestinal histology for comparison and further research is needed. Several studies have reported an increase in villus dimension in broilers in response to phytobiotics. These positive effects have been attributed to their antiparasitic, antioxidant, and antimicrobial properties (Khan et al. 2023). The enlargement of villus dimensions is believed to enhance the digestive and absorptive capacities of the gut by providing a larger surface area for absorption. However, in the current study, no discernible effects of HS on histological dimensions were observed. Further research is warranted to elucidate the impact of HS on histological features. In the current study, a significant increase in WBCs count, as well as IgG and IgM antibody titres on day 28, was observed in the HS-20 group compared to the control. The available literature on the impact of HS on the immune system in animal studies is limited, underscoring the need for further research to elucidate the mechanisms through which cannabis exerts its immunomodulatory effects. Enhanced immune responses have been achieved in studies by Afzali et al. (2015) and Barani et al. (2015) involving broilers fed extruded HS. Conversely, these authors discovered that chicken groups fed diets containing varying ratios of HS and processed HS extrudates displayed elevated immunoglobulin G titers in comparison to the control group. Rezapour-Firouzi et al. (2018) reported improved immune response in human subject fed with HS. In addition to essential nutrients, cannabis contains compounds like plant sterols and phytocannabinoids, which encompass cannabinoids exclusively present in cannabis. With over 60 phyto-cannabinoids, cannabis exhibits anti-inflammatory, antimicrobial, immunomodulatory, and antioxidant properties (Stastník et al. 2019). Some researchers propose that HS stands out as a significant protein source, characterized by its rich arginine content, which has been correlated with promoting an optimal immune response (Farinon et al. 2020). Cannabinoids interact with two receptors known as CB1 and CB2, which have been identified in all animals (Begg et al. 2005). CB1 receptors are primarily located in the brain and are present in the reproductive systems. On the other hand, CB2 receptors are predominantly found in the immune system, with the highest concentration in the spleen. This receptor is believed to be responsible for the anti-inflammatory effects and potentially other therapeutic benefits of cannabis (Núñez et al. 2004). Similar to our study, Mahmoudi et al. (2015) reported no significant effect of different levels of HS (25, 50 and 75%) on antibody titre of antibody production, blood count and relative weight of bursa and spleen in broilers. The observed significant increase in white blood cell count, as well as IgG and IgM antibody titres on day 28 in the HS-20 group compared to the control, highlights a crucial finding in the study regarding the enhancement of immune parameters. This finding is of paramount importance as it indicates that the supplementation of HS positively impacts the immune response of broiler chicks. In practice, such improvement in immune parameters translates to healthier birds that are more resistant to various stressors and diseases. With enhanced immune function, there may be reduced reliance on antibiotic usage, contributing to better animal welfare and addressing concerns regarding antibiotic resistance. Moreover, poultry products derived from birds with bolstered immune systems may garner higher consumer acceptance due to perceived health benefits, thus benefiting both producers and consumers alike.

## Conclusion

In conclusion, the obtained results indicated that supplementation of HS in broiler diet could markedly improve immune responses without any adverse effects on growth performance and gut of broilers.

## Data Availability

The data are available on request.
